# Implementation of a hospital antibiotic stewardship program: first results

**DOI:** 10.1186/2047-2994-4-S1-P180

**Published:** 2015-06-16

**Authors:** I Neves, V Alves, A Duraes, R Correia Abreu, S Jordão, M Guimaraes, MJ Soares, D Peres, F Vieira, I Devesa, Antibiotic Stewardship Group

**Affiliations:** 1 Infectious Diseases Unit, Unidade Local de Saúde de Matosinhos., Matosinhos, Portugal; 2 Microbiology Laboratory, Unidade Local de Saúde de Matosinhos., Matosinhos, Portugal; 3 Pharmacy, Unidade Local de Saúde de Matosinhos., Matosinhos, Portugal; 4 Infection Control and Antimicrobial Resistance Unit, Unidade Local de Saúde de Matosinhos., Matosinhos, Portugal

## Introduction

Portugal has high rates of healthcare associated infection and antimicrobial resistance. In February of 2013 the National Program on Prevention and Infection Control and Antimicrobial Resistance (PPCIRA) was restructured. In November of 2013 the PPCIRA determined implementation of Antibiotic Stewardship Programs (ASP) in all healthcare facilities.

## Objectives

Implementation of a ASP, with mandatory validation of all the prescriptions of carbapenems and quinolones in the first 96 hours, medical education, carbapenems and quinolones consumption reduction and increase of antibiotic free days.

## Methods

Establishment of Antibiotic Stewardship Group in a 400 bed acute care hospital, consisting of Infectious Diseases specialists, who advise and validate the prescription, and Microbiologists, with consulting function. A database network was built to monitor the prescription of carbapenems and quinolones, with alerts sent via email to the group. This database was linked to a validation system, allowing monitoring, auditing and information to the prescriber. The result of the audit appears in warning messages when opening the prescription (adequate or inadequate). If it’s “inadequate”, the infectious diseases specialists contacts prescribing physician for advice. The ASP began in October 2014 and had the involvement of the institutional leaderships.

## Results

During the first 3 months of the implementation of ASP the prescriptions decreased 38% and the seeking of advice pre-antibiotic prescription increased. From 2013 to 2014 there was a decrease in the consumption of quinolones from 41,5 to 36,9 DDD/100 patients and of carbapenems from 47,8 to 46,3, respectively. The antibiotic free days increased from 43,5 in 2013 to 50,1 in 2014.

## Conclusion

Implementation of a ASP in an acute care hospital allowed reduction of consumption of carbapenems and quinolones, as well as an increase of antibiotic free days. Its success is dependent on the involvement of the leadership, coordinated multidisciplinary approaches and education.

## Disclosure of interest

None declared.

